# Modelling individual differences in reading using an optimised MikeNet simulator: the impact of reading instruction

**DOI:** 10.3389/fnhum.2024.1356483

**Published:** 2024-06-21

**Authors:** Ya-Ning Chang, Ting-Jung Chang, Wei-Fen Lin, Ching-En Kuo, Yu-Ting Shi, Hung-Wei Lee

**Affiliations:** ^1^Miin Wu School of Computing, National Cheng Kung University, Tainan, Taiwan; ^2^Department of Computer Science, National Yang-Ming Chiao-Tung University, Hsinchu, Taiwan

**Keywords:** individual differences, word reading, computational modelling, reading instruction, semantic reliance

## Abstract

Reading is vital for acquiring knowledge and studies have demonstrated that phonology-focused interventions generally yield greater improvements than meaning-focused interventions in English among children with reading disabilities. However, the effectiveness of reading instruction can vary among individuals. Among the various factors that impact reading skills like reading exposure and oral language skills, reading instruction is critical in facilitating children’s development into skilled readers; it can significantly influence reading strategies, and contribute to individual differences in reading. To investigate this assumption, we developed a computational model of reading with an optimised MikeNet simulator. In keeping with educational practices, the model underwent training with three different instructional methods: phonology-focused training, meaning-focused training, and phonology-meaning balanced training. We used semantic reliance (SR), a measure of the relative reliance on print-to-sound and print-to-meaning mappings under the different training conditions in the model, as an indicator of individual differences in reading. The simulation results demonstrated a direct link between SR levels and the type of reading instruction. Additionally, the SR scores were able to predict model performance in reading-aloud tasks: higher SR scores were correlated with increased phonological errors and reduced phonological activation. These findings are consistent with data from both behavioral and neuroimaging studies and offer insights into the impact of instructional methods on reading behaviors, while revealing individual differences in reading and the importance of integrating OP and OS instruction approaches for beginning readers.

## 1 Introduction

Reading is an acquired skill that enables people to absorb ideas through written languages. To become a proficient reader, children need to learn the mappings between orthography (O), semantics (S), and phonology (P). Past literature has shown that reading behaviors differ across readers as a function of their sensitivity to OP and OS mappings during reading (Hoffman et al., 2015; Woollams et al., 2016; Siegelman et al., 2020, 2022). Moreover, these individual differences may be critical factors that influence the effectiveness of reading instruction. For example, in designing and implementing intervention programs for children with reading disabilities (RD), Siegelman et al. (2022) demonstrated significant intervention gains through phonology-focused instruction for RD children that relied more on OP regularities than OS regularities for reading. Hence, the differences in sensitivity to OP and OS mapping in reading performance motivated the present study to examine factors influencing beginning readers’ sensitivity to different regularities.

In reading literature, several factors have been proposed to contribute to individual differences in reading, such as reading experience (Yap et al., 2012; Andrews, 2015), reading capacity (Plaut, 1997; Dilkina et al., 2008), and oral language skills (Siegelman et al., 2020; Chang, 2023). Additionally, early reading training (Taylor et al., 2017; Chang et al., 2020) could be critical concerning children’s sensitivity to OP and OS regularities. However, whether there is a direct link between them remains unclear. Consequently, in this study, we utilised a series of well-established computational models of reading trained with three different types of instructional methods including phonology-focused training, meaning-focused training, and phonology-meaning balanced training. The investigation aimed to determine the models’ sensitivity to OP and OS regularities by drawing on a measure of semantic reliance (SR hereafter) to operationalise as the utilisation of OP and OS pathways. We then compared model performance with behavioral data reported in the reading literature (Woollams et al., 2016; Siegelman et al., 2020, 2022). Here, we provided a brief overview of individual differences in reading, early reading instruction and the computational framework employed in our model development.

### 1.1 Individual differences in reading

Studies have investigated the variability in reading behaviors among typical readers (Hoffman et al., 2015; Woollams et al., 2016; Davies et al., 2017; Siegelman et al., 2020, 2022; Chang, 2023) and dyslexic readers (Ziegler et al., 2008; Perry et al., 2019). Particularly, inter-subject variability in efficient OP and OS mapping sensitivity has been considered critical in contributing to variations in reading performance (Hoffman et al., 2015; Woollams et al., 2016; Siegelman et al., 2020, 2022). For instance, Woollams et al. (2016) examined how individual differences in the degree to which readers rely on semantic access affect reading performances in a reading-aloud task. They demonstrated that skilled readers with high SR tend to show a stronger imageability effect and produce slower responses than those with low SR particularly when reading words with inconsistent spelling-to-sound mappings (e.g., *pint*) in a reading-aloud task. Comparable variations in reading behaviors among individuals are also evident in research involving children (Siegelman et al., 2020, 2022). In a recent study involving a large cohort of children, it was observed that children demonstrating high sensitivity to OP regularities generally outperformed their counterparts in reading tasks, as opposed to those with lower sensitivity to OP regularities (Siegelman et al., 2020). Additionally, these children tended to show substantial gains in intervention outcomes (Siegelman et al., 2022). These findings suggest that children’s sensitivity to both OP and OS regularities may play a pivotal role in shaping their reading performance and the effectiveness of reading interventions.

As noted earlier, a number of factors may contribute to individual differences in adults and children. Critical factors, including reading experience (Yap et al., 2012; Andrews, 2015), reading capacity (Plaut, 1997; Dilkina et al., 2008), and oral language skills (Siegelman et al., 2020; Chang, 2023), have been proposed and investigated. Overall, the findings suggest that individuals with extensive reading experience, efficient processing in the reading system, and strong oral language skills typically can develop high-quality orthographic, phonological, and semantic representations, as well as efficient reading pathways (connections between these representations). Besides these factors, early reading training (Taylor et al., 2017; Chang et al., 2020) may also play a crucial role in influencing readers’ sensitivity to OP and OS regularities. However, the extent to which early reading training directly correlates with OP and OS mapping sensitivity remains uncertain.

### 1.2 Early reading instruction

For decades, the effectiveness of early reading instruction has been a prominent topic in English reading literature (Rayner et al., 2001; Nation, 2009; Suggate, 2016; Taylor et al., 2017; Castles et al., 2018; Torgerson et al., 2019). When it comes to learning to read in English, there are two contrasting types of instructional methods. One is phonology-focused training (e.g., Bus and van Ijzendoorn, 1999; Ehri et al., 2001), in which children are instructed to learn intensively about the relationships between print and sound. The other is meaning-focused training or a whole language approach (Levy and Lysynchuk, 1997), in which children are instructed to learn intensively about the relationships between whole words and their meanings using word cards with or without accompanying pictures.

Phonology-focused training can be traced back to the characteristics of the English writing system. There are more consistent mappings between spelling and sound compared to mappings between spelling and meaning, particularly for words comprising a single morpheme (i.e., monomorphemic words) (Plaut and Gonnerman, 2000). Additionally, monomorphemic words are frequently encountered in the early reading stages (Rastle, 2019). As a result, phonology-focused instruction can assist children in leveraging the systematic relationships between letters and sounds (e.g., *-int* in *mint* and *hint*), making it a more accessible learning approach. Moreover, in line with the self-teaching hypothesis (Share, 1995), the ability to map spelling to sound serves as a critical self-teaching mechanism for children. This skill aids in the subsequent acquisition of rapid and detailed orthographic representations of novel words and facilitates their recognition. Multiple lines of evidence from behavioral, neuroimaging and computational studies (Taylor et al., 2017; Chang et al., 2020) have demonstrated the effectiveness of phonology-focused training not only in word reading but also in tasks related to word comprehension. Taylor et al. (2017) conducted both behavioral and neuroimaging assessments, revealing that individuals undergoing phonology-focused training exhibited notable benefits in terms of accuracy and speed in a reading-aloud task compared to those who received meaning-focused training. Moreover, comparable performance between the two training methods was observed in a reading comprehension task. A subsequent modelling study by Chang et al. (2020) further highlighted that this transfer effect was largely dependent on oral language skills in addition to spelling-to-sound skills. Collectively, these findings align with the framework of the Simple View of Reading (SVR) (Gough and Tunmer, 1986), suggesting that successful reading comprehension necessitates a combination of phonological decoding and oral language skills.

Conversely, advocates of meaning-focused training put forth two arguments in support of their perspective (e.g., Davis, 2013). Firstly, they emphasise that the ultimate objective of reading is to establish a direct connection between the spelling of a word and its meaning, warranting a thorough examination of the efficacy of this direct mapping approach. Secondly, they point out the presence of morphological regularities in English, especially for words consisting of more than one morpheme, as exemplified by pairs like *bake* and *baker*. Research, as indicated by Nation and Cocksey (2009), suggests that children can access meaning from an orthography system without the need for phonological involvement. Consequently, proponents of meaning-focused training propose that it is beneficial for children to acquire OS mappings early on in reading (Levy and Lysynchuk, 1997; Nation, 2009; Taylor et al., 2015). However, as pointed out by Share (1995), meaning-focused training faces a noteworthy hurdle in providing a practical acquisition strategy for obtaining orthographic representations of unfamiliar words and, consequently, in navigating through an orthographic avalanche. This becomes particularly challenging considering that, on average, a fifth grader is exposed to approximately 10,000 new words in natural printed text (Nagy and Herman, 1987). It is important then that current educational practice integrates OP and OS strategies in early reading instruction and intervention.

The phonology-focused training and meaning-focused training are in accordance with neurocomputational models of dual language and reading pathways (Hickok and Poeppel, 2004, 2007; Rauschecker and Scott, 2009; Ueno et al., 2011; Bornkessel-Schlesewsky and Schlesewsky, 2013). For reading, a *dorsal* pathway generally consisting of the fusiform gyrus, inferior supramarginal, premotor cortex, and inferior frontal gyrus (pars opercularis and pars triangularis) underpins print-to-sound processes, while a *ventral* pathway generally consisting of the fusiform gyrus, the anterior parts of middle and superior temporal gyrus, the anterior temporal pole, and the inferior frontal gyrus (orbitalis) underpins print-to-meaning processes (Price, 2012; Carreiras et al., 2014, for a review; Taylor et al., 2013; Hoffman et al., 2015). Additionally, several studies have investigated neural activities of learning to read. It is found that modulation of dorsal pathway activity is evident when learning print-to-sound associations of new words while modulation of ventral pathway activity is elusive when learning whole object or word names (Mei et al., 2014; Quinn et al., 2017; Taylor et al., 2017). Particularly, a recent neuroimaging by Taylor et al. (2017) investigates the neural consequences of reading instruction (phonology-focused training versus meaning-focused training). The result demonstrated that meaning-focused training increased neural effort in dorsal pathway regions compared to phonology-focused training; however, there were no differences in ventral pathway activity following the two instructional approaches. Importantly, the fMRI results from Taylor et al. (2017) showed striking benefits of print-to-sound training. The authors suggest that early literacy education in alphabetic languages should focus on OP rather than OS strategies in order to enhance both reading aloud and reading comprehension.

While phonology-focused training and meaning-focused training represent distinct reading instructional approaches, they are frequently integrated to some extent in practical applications. Consequently, assessing their effectiveness and their influence on subsequent reading behaviors can be challenging.^1^ However, as evidence accumulates, a growing consensus in reading research suggests that phonology-focused training is crucial for acquiring the skill to read words with a semi-transparent relationship between print and sound in English, especially in the early stages of reading instruction (Castles et al., 2018, for a review; Rastle, 2019), compared to meaning-focused training. Nevertheless, research into the impact of these different types of reading instructional methods on OP and OS mapping sensitivity is still ongoing.

### 1.3 The computational model of reading

Progress in the computational modelling of reading has offered valuable mechanistic explanations for general processing principles within the human reading system. These models simulate human reading performance across diverse populations including children, adults and patients (Seidenberg and McClelland, 1989; Plaut et al., 1996; Harm and Seidenberg, 1999, 2004; Coltheart et al., 2001; Harm et al., 2003; Powell et al., 2006; Ziegler et al., 2008; Perry et al., 2019). Within the triangle modelling framework (Seidenberg and McClelland, 1989; Plaut et al., 1996; Harm and Seidenberg, 2004), the process of learning to read can be achieved through various pathways, depending on the division of labour along direct and indirect pathways for accessing phonology or semantics from orthography. This inherent property of the triangle model positions it as an ideal platform for investigating OP and OS mapping sensitivity in reading. Our previous modelling research, based on the triangle modelling framework, implemented different early reading instructional schemes used in the behavioral study by Taylor et al. (2017). The simulation results demonstrated the importance of oral language skills on the effectiveness of reading instruction (Chang et al., 2020). However, the simulation study did not examine how early reading instruction may contribute to individual differences in reading, particularly regarding the utilisation of OP and OS pathways in the model and its potential impact on later mature reading behaviors.

Therefore, building upon our prior modelling research (Chang et al., 2020), the current study aimed to address these challenges. Specifically, following the educational approaches reviewed in section 1.2, two contrasting early reading training schemes were implemented, each focusing on a different aspect of reading instructional methods: OP-focused and OS-focused. Based on previous behavioral and modelling studies (Taylor et al., 2017; Chang et al., 2020), the OP-focused training model underwent three times the training on OP mappings, while the OS-focused training model received three times the training on OS mappings. Considering that OP and OS training are also frequently integrated to some extent in practical applications, we implemented a mixture of OP and OS training models. As there is no specific guidance on the proportion of mixture, the model was trained with an equal amount of training on both OP and OS mappings, termed the OP-OS balanced model. Following Chang (2023), the utilisation of OP and OS pathways was characterised by SR in the model as an indicator of individual differences. Subsequently, we explored the associations between the SR in the model and various early training regimens. Additionally, we examined how SR interacted with other psycholinguistic reading effects during reading aloud.

Finally, while computational modelling has proven to be a valuable tool for probing the mechanisms underlying language processes, training a large-scale deep neural network model, such as the fully implemented triangle model of reading employed in this study, is often associated with high computational costs. Consequently, the time-consuming training process fundamentally limits the model’s capacity to simulate a substantial cohort of individuals. To mitigate the training burden and facilitate large-scale of computational studies, optimising and parallelising the computationally demanding algorithms is imperative by leveraging available computing hardware resources. In this study, we integrated Single Instruction Multiple Data (SIMD) and threading optimisation techniques to enhance the computational performance and efficiency. Further details about the optimisation approaches and results are reported in the Supplementary material.

## 2 Method

### 2.1 Model architecture

**Figure 1:**
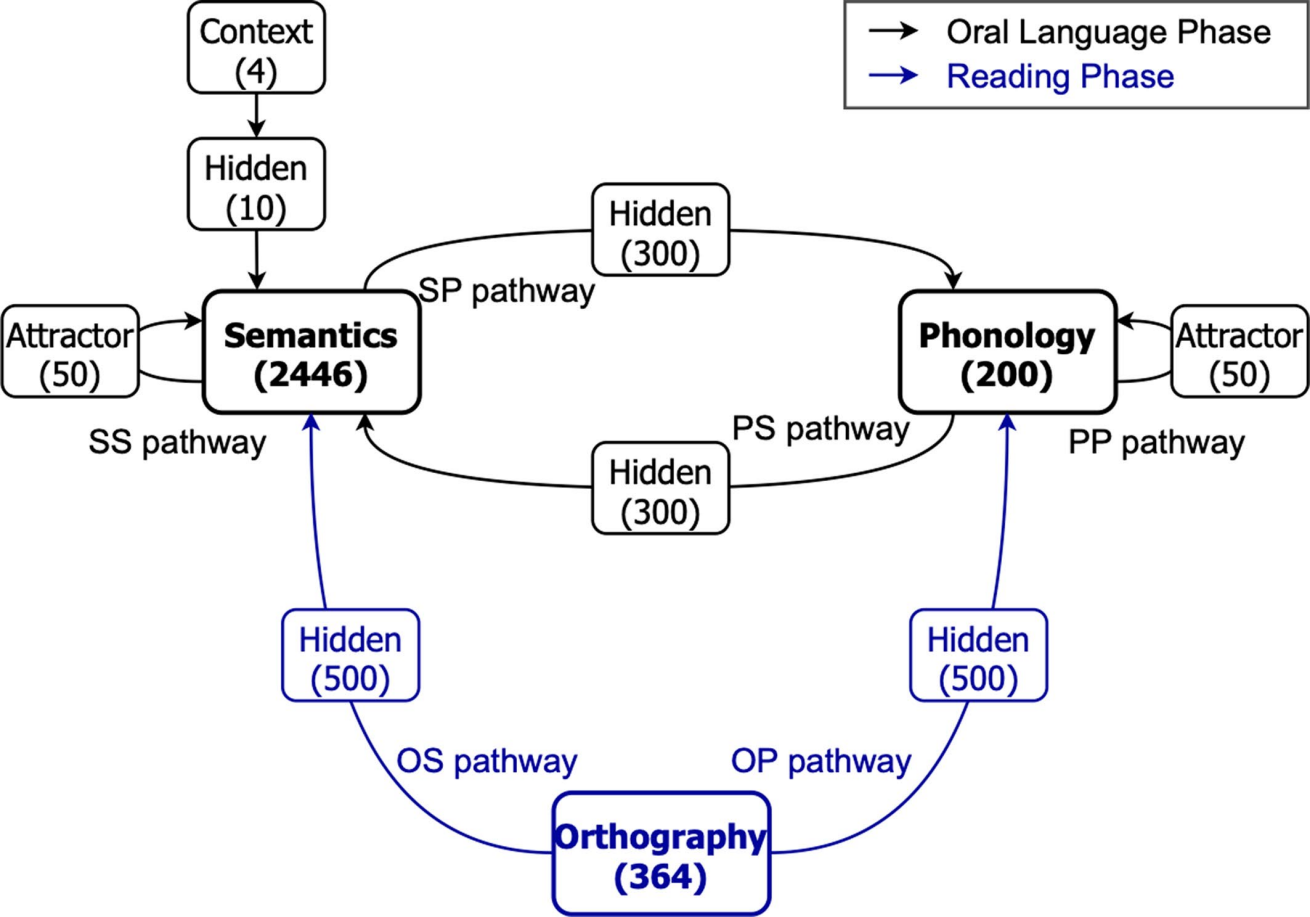
The architecture of the triangle model of reading. Numbers in brackets indicate the number of units in that layer. S: Semantics; P: Phonology; O: Orthography. Adapted from Chang (2023).

Figure 1 shows the architecture of the triangle model of reading. The model was identical to the one used in previous modelling studies exploring the influence of oral language skills on variations in reading (Chang, 2023), the effectiveness of reading instruction (Chang et al., 2020), as well as investigating the impact of word sequence and language exposure on reading development (Monaghan et al., 2017; Chang and Monaghan, 2019). It had three essential processing layers including orthography (O), phonology (P), and semantics (S). There were 364 units in the orthography layer, 200 units in the phonology layer, and 2,446 units in the semantics layer. All three layers connected to each other but between every two layers was a hidden layer. The hidden layers between the phonological and semantic layers had 300 units, while those between the orthographic, phonological and semantic layers had 500 units. In phonological and semantic layers, there were attractor layers consisting of 50 units. These attractor layers helped the model develop robust and accurate phonological and semantic representations of words, wherein even partial or noisy degraded activation patterns could transition towards familiar representations (Harm and Seidenberg, 2004). Moreover, a context layer consisting of 4 units was connected to the semantic layer via a hidden layer of 10 units to handle homophones (i.e., words have the same orthographic form but multiple meanings).

### 2.2 Word representations

Following previous modelling work (Chang et al., 2019; Chang and Monaghan, 2019; Chang, 2023), the model was trained with orthographic, phonological, and semantic patterns for 6,229 monosyllabic English words. This set of vocabulary encompassed most of the inflected forms found in monosyllabic English words (Harm and Seidenberg, 2004). Orthographic patterns of words were represented by 14 letter slots, each containing 26 units corresponding to the English alphabet. The alignment of words involved situating the first vowel letter on the fifth slot, and if applicable, the second vowel letter on the sixth slot. Consonant letters that preceded or followed the vowel letters were placed in slots adjacent to the two vowel letter slots. For example, *yes* was represented as _ _ _ y e _ s _ _ _ _ _ _ _, and *great* as _ _ g r e a t _ _ _ _ _ _ _.

Phonological patterns of words were represented by 8 phoneme slots, each comprising 25 units for various phonological features such as voiced, nasal, round, etc. The first three phoneme slots were designated for onset consonants, the fourth slot for the vowel, and the remaining four slots for coda consonants. For example, *yes* was _ _ y E s _ _ _ and *great* was _ g r eI t _ _ _.

Semantic representations of words were derived from Wordnet (Miller et al., 1990) and consisted of 2,446 semantic units. The representational scheme employed binary coding, where one indicated the presence of a semantic feature, and zero indicated its absence. Context representations for each meaning of the homophone family were randomly assigned by activating one of the four units at the beginning of training. None of the context units were active for words with a single meaning.

### 2.3 Training procedure

#### 2.3.1 Model training

The procedure for model training was divided into two phases: the oral language training phase, which mimicked children’s learning to listen and speak, and the reading training phase, which mimicked children’s learning to read. For each training trial, the model was presented with a word randomly chosen according to its word frequency (Marcus et al., 1993).

In the oral language phase, the model was trained with four oral language tasks interleaved for two million trials. For each trial, one of the four mappings, semantics to phonology (SP), phonology to semantics (PS), phonology to phonology (PP), or semantics to semantics (SS) was selected for training. The PS and SP mappings in the model were used to simulate an oral vocabulary task and a meaning naming task in the human behavioral studies, respectively. On the other hand, the PP and SS mappings were used for the model to develop a reliable phonological attractor and a semantic attractor, respectively. The training ratio was 40% of trials for SP, 40% of trials for PS, 10% of trials for PP and 10% of trials for SS. For the SP training, the model was provided with an input to the semantic layer for eight timesteps, and the model was asked to generate the corresponding target in the phonological layer. Similarly, for the PS training, the model was provided with an input to the phonological layer for eight timesteps, and the model was asked to generate the corresponding target in the semantic layer. For the phonological attractor, the phonological representations were presented for two timesteps and the model was allowed to cycle the activation for the next six timesteps to recreate initial representations. Likewise, the semantic attractor was to map semantic to semantic representations, and the timesteps for presentation and cycling were identical to those in training the phonological attractor.

In the reading training phase, all weights between the phonological and semantic layers obtained from the oral language training phase were first loaded and frozen. The model was then trained to learn to read by learning the mappings from orthographic to phonological representations and from orthographic to semantic representations for one million trials. A word was selected and presented for twelve timesteps in each trial. Critically, three different early training schemes, OP-focused training, OS-focused training, and OP-OS balanced training were implemented by varying amounts of exposure to OP or OS mapping following previous behavioral and modelling studies (Taylor et al., 2017; Chang et al., 2020). Specifically, for OP-focused training, the probability of exposure to OP mapping was 75%, and the probability of exposure to OS mapping was 25% (i.e., OP exposure was 3x higher than OS exposure). In contrast, for OS-focused training, the probability of exposure to OP mapping was 25%, and the probability of exposure to OS mapping was 75%. For OP-OS balanced training, the probability of exposure to the OP or OS exposure was equal, both 50% (i.e., identical mapping exposure).

In both phases of model training, the same training parameters were employed. The backpropagation through time algorithm (Pearlmutter, 1989, 1995) was used to update weights by reducing the differences between target patterns and actual activations produced by the model. The learning rate was set to 0.05. To simulate variability in each reading training condition, 40 versions of the OP-focused model, the OS-focused model, and the OP-OS balanced model were trained separately with different initial weights. Thus, there were in total 120 simulations of the models.

### 2.4 Testing procedure

After the end of model training, the evaluation of model performance followed the methodology established in prior simulation work (Chang and Monaghan, 2019; Chang, 2023). In assessing phonology, the error score was computed as the sum of squared error (SSE) between the model’s actual phonological pattern and its target pattern. Accuracy was determined by identifying the closest phoneme to the model’s output through Euclidean distance and verifying whether the actual and target phonemes matched across all phoneme slots. Similar approaches were used to assess semantics. The error score was calculated as the semantic SSE between the model’s actual semantic pattern and its target pattern. Accuracy, in this context, involved computing the Euclidean distance between the model’s actual semantic representation and the semantic representation of each word in the training set. The assessment then determined if the smallest distance corresponded to the target semantic representation.

### 2.5 Measuring semantic reliance in the model

A crucial aim of this study was to explore whether training focus would influence individual reading strategies in the model, in terms of the degree of utilisation of OP and OS pathways reflected by the measure of SR. As demonstrated in a recent simulation study by Chang (2023), the SR in the model could be quantified by directly measuring the use of the semantic pathway relative to the phonological pathway using a lesion technique (Welbourne et al., 2011; Chang and Lambon Ralph, 2020), or by indirectly measuring the consistency effect when reading exception words, as used in a behavioral study by Woollams et al. (2016). Critically, investigations into these two SR measures resulted in largely similar patterns regarding their predictiveness on reading performance, albeit predictability was greater for the direct approach than the indirect one.

Therefore, in the present study, the direct approach was adopted to measure SR in the model. Specifically, to quantify the use of the OS pathway in accessing semantics in the model, the OPS pathway was damaged, and semantic SSE was recorded. The reverse procedure was used to determine the use of the OPS pathway by damaging the OS pathway. The assumption is that if one pathway is damaged, and the model relies on the undamaged pathway, resulting in minimal semantic SSEs, it indicates effective functioning and high reliability of the undamaged pathway. Conversely, if reliance on the undamaged pathway leads to a substantial semantic SSE, it suggests poor functioning of the undamaged pathway. Consequently, the reciprocals of the semantic SSE obtained from the OS and OPS pathways were computed to denote the proportional contribution across the pathways. The SR was quantified by dividing the contribution of the OS pathway by the sum of the contributions from the OPS and OS pathways.

## 3 Results

### 3.1 Model performance

At the end of the oral language training, the model achieved 92.11% accuracy on the meaning naming task (which required SP mappings) and 91.24% on the oral vocabulary task (which required PS mappings). At the end of the reading training, the OP-focused model, the OP-OS balanced model, and the OS-focused model achieved an accuracy of 99.83, 99.96, and 99.23% in phonology, and an accuracy of 92.61, 99.43, and 98.91% in semantics, respectively.

### 3.2 Individual-level analysis: the relationship between reading instruction and the SR

**Figure 2:**
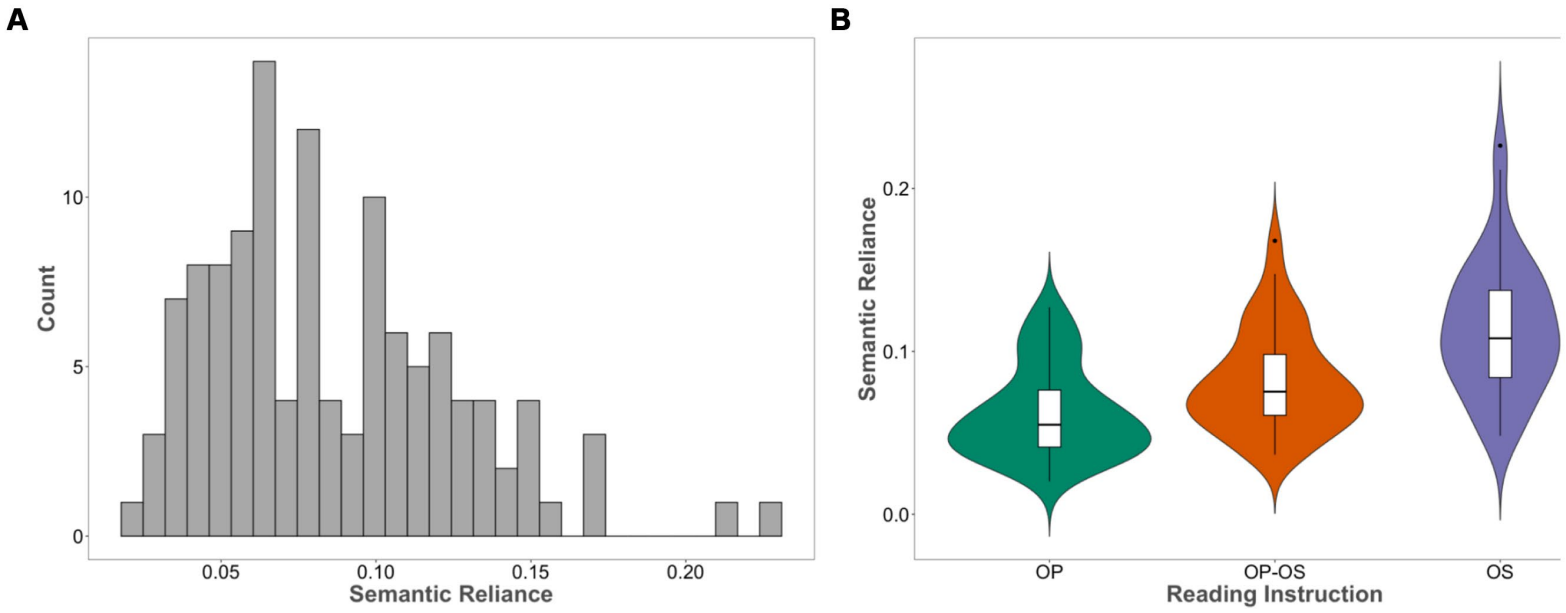
**(A)** The distribution of the semantic reliance in the model **(B)** the semantic reliance generated from the models with different types of reading instructional approaches. OP: OP-focused model; OP-OS: OP-OS balanced; OS: OS-focused.

The SR was calculated for each of the 120 models including 40 versions of the OP-focused model, 40 versions of the OP-OS balanced model, and 40 versions of the OS-focused model. Figure 2A illustrates the SR distribution, while Figure 2B presents the SR scores corresponding to each type of reading instruction. The SR ranged from 0.02 to 0.226 (*M* = 0.086, SD = 0.040), and the average SR was the highest for the OS-focused model (*M* = 0.114, SD = 0.041), followed by the OP-OS balanced model (*M* = 0.081, SD = 0.031), and then the OP-focused model (*M* = 0.062, SD = 0.028). The SR distribution demonstrated that varying training environments could lead to different degrees of SR in the model of reading.

The relationship between reading instruction and SR was directly investigated using a simple regression technique with training focus, OP-focused (OP), OP-OS balanced (OP-OS), and OS-focused (OS) as the predictor and SR as the dependent variable. The regression model was significant, in which *R*^2^ value was 29.5% (Adjusted *R*^2^ = 28.3%), *p* < 0.001. The SR difference between the OP model and the OP-OS model was significant, *β* = 0.227, *p* = 0.013. The SR differences between the OP-OS model and the OS model, *β* = 0.393, *p* < 0.001, and between the OP model and the OS model, *β* = 0.62, *p* < 0.001, were both significant.

The regression results showed that training focus could explain a significant portion of the variance in SR, demonstrating the influence of training focus on the utilisation of OP and OS pathways in the model. To further explore the extent to which training focus might impact learning in terms of the development of precise representations in the model and its relationship with SR, we investigated the level of activations among units at the phonological layer, referred to as *stress* or *polarity* (Plaut, 1997). The concept of polarity measurement suggests that during training, units at the output layers of the model are trained to accurately represent target patterns typically consisting of binary values (i.e., 1 or 0). Consequently, higher polarity scores generated by the model for a given word indicate the development of more refined representations for that word. Following Plaut (1997), we used a formula (Equation 1) to compute the index of unit binarisation in the model, termed unit polarity:


(1) $$y=x*\log_{2}\left(x\right)+\left(1-x\right)*\log_{2}\left(1-x\right)+1$$

where x is the unit activation ranging from 0 to 1; $\log_{2}\left(\right)$ is the logarithmic function with the base of 2; y is the polarity measure.

**Figure 3:**
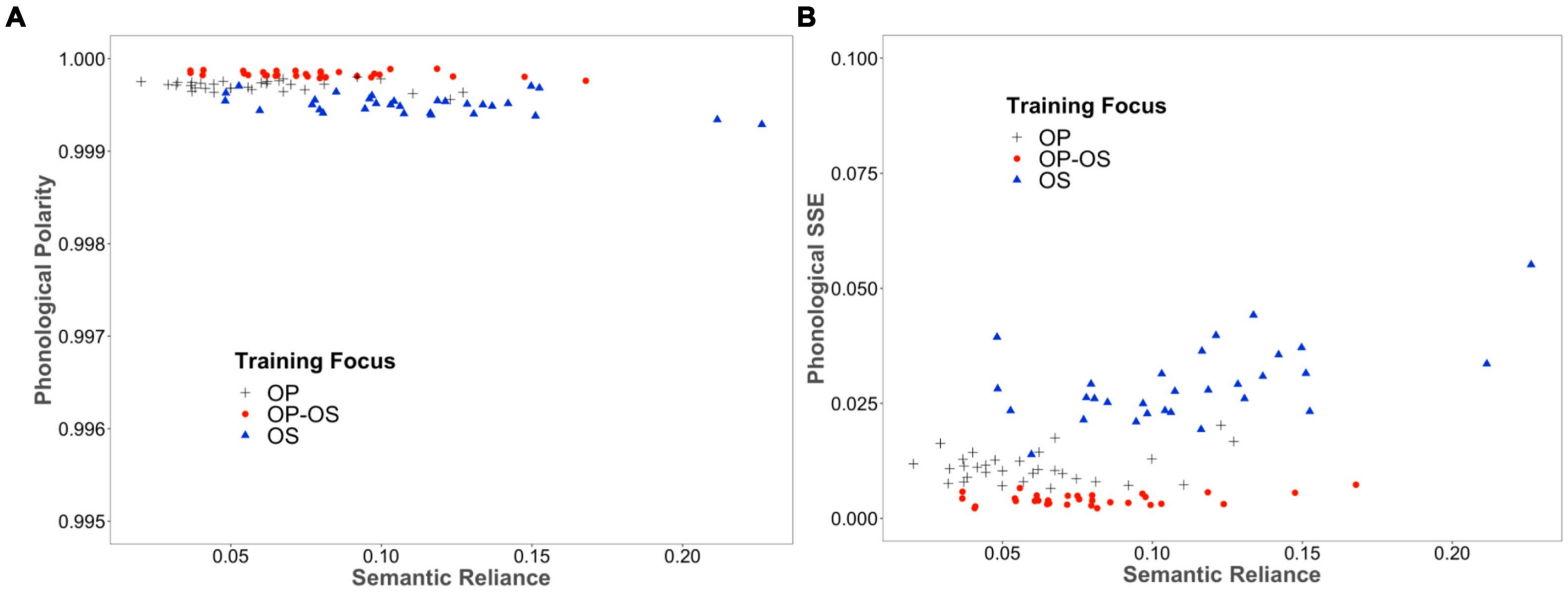
**(A)** The phonological polarity **(B)** the phonological SSE was generated by each of 120 individual models, categorised by varying SR scores and different training focuses.

We computed the average of polarities across all words in the training set at the phonological layer for each model, categorised by varying SR scores and different training focuses. The result is illustrated in Figure 3A. As can be seen, both polarity scores generated by the OP-focused models and OP-OS balanced models are higher than those generated by the OS-focused models. Critically, these scores are further modulated by SR, with an increase in SR resulting in a decrease in polarity scores especially for the OS-focused models. In Figure 3B, we also showed the corresponding phonological SSE for each model, confirming that the models that produced higher polarity scores tended to have smaller phonological SSEs, reflecting more refined representations.

### 3.3 Exploring the influence of SR on reading performance of the model

The following critical examination in this study was to explore whether the SR influenced by varying training focuses could impact reading behaviors in the model. Past behavioral investigations have shown that SR affects RTs in a reading-aloud task for both adults (Woollams et al., 2016) and children (Siegelman et al., 2022). Particularly, skilled readers with high SR tend to produce slower responses than readers with low SR; and their RTs are moderated by imageability and consistency. Therefore, to investigate whether the present model could produce similar patterns as observed in behavioral studies, we conducted a series of linear mixed-effect model (LMM) analyses on the model’s reading-aloud performance. Phonological SSE in the model was used as a proxy for human RTs in a reading-aloud task (Monaghan et al., 2017; Chang et al., 2020). LMM analyses were based on “lme4”, a package in R (version 4.1.3, 2022). For the LMM analyses, an effect was considered significant at the *p* < 0.05 level if its t-value was greater than 1.96 (Baayen, 2008). To examine the SR effect and its relationship with other psycholinguistic variables, we conducted two evaluation approaches, one was to replicate the findings reported in a factorial experiment reported by Woollams et al. (2016), and the other one was to examine the effect using a regression-based approach.

#### 3.3.1 The factorial test of the SR effect

For the factorial test, we investigated whether the model could replicate the key behavioral patterns of the reading-aloud task observed in Woollams et al. (2016), wherein the SR effect is modulated by imageability and consistency. Four sets of stimuli, including high-imageability consistent words, low-imageability consistent words, high-imageability inconsistent words, and low-imageability inconsistent words were taken from Woollams et al. (2016), comprising 40 words for each condition. There was a total of 19,200 trials (i.e., 4*40*120). Prior to the analysis, 6.7% of the trials including words misread by the model and phonological SSEs greater than three standard deviations from the mean, were discarded as outliers. This resulted in 17,912 data points for further analysis. An LMM model was conducted with item and model version as random factors, with imageability by consistency by SR as fixed factors, and with phonological SSE as the dependent variable. All variables were scaled using z-score normalisation for evaluating interactions. The results showed that SR significantly predicted phonological SSE, *β* = 0.005, *t* = 3.72, as well as consistency, *β* = −0.004, *t* = −2.51. Imageability was not a significant predictor. The interaction between SR and consistency, *β* = −0.002, *t* = −4.13, was significant. The interaction between SR and imageability, *β* = −0.002, *t* = −2.02 was also significant. The interaction between consistency and imageability was not significant. Critically, the three-way interaction between SR, imageability, and consistency was significant, *β* = −0.001, *t* = −2.41.

#### 3.3.2 The regression-based test of the SR effect

For the test using the regression-based approach, in addition to SR, we included a range of psycholinguistic variables previously shown to be important for the reading-aloud task in behavioral studies: word frequency (WF), orthographic neighbourhood size (ONS) (Coltheart et al., 1977), rime consistency (RC) (Glushko, 1979), and imageability (IMG) (Cortese and Fugett, 2004). The descriptive statistics of the psycholinguistic variables and the correlations between them are reported in Table 1.

**Table 1:** The descriptive statistics of the psycholinguistic variables and the correlations between them.

	WF	ONS	RC	IMG
WF (*M* = 0.34, SD = 0.31)	1			
ONS (*M* = 6.11, SD = 5.17)	0.18^***^	1		
RC (*M* = 0.92, SD = 0.20)	−0.12^***^	−0.01	1	
IMG (*M* = 4.26, SD = 1.40)	0.18^***^	0.09^***^	0.02	1

^***^p < 0.001; ^**^p < 0.01; ^*^p < 0.05; WF: word frequency; ONS: orthographic neighbourhood size. RC: rime consistency; IMG: imageability. M: Mean, SD: standard deviation.

Data cleaning procedures were similar to those in the previous section. Outliers included words that the model misread, had missing psycholinguistic measures, and phonological SSE greater than three standard deviations from the means. This removal resulted in 590,653 points. To explore whether the SR effect was moderated by imageability and consistency, an LMM analysis was conducted with all the psycholinguistic variables and the interaction between SR, imageability and consistency as independent variables, and with phonological SSE as a dependent variable. As shown in Table 2, WF, ONS, RC, and IMG all had significant effects on the model’s reading-aloud performance. Specifically, the model produced low phonological SSEs for words with higher values of WF, ONS, RC, and IMG, congruent with previous findings in behavioral studies (Balota et al., 2004; Cortese and Khanna, 2008). Additionally, SR was also a significant predictor, *β =* 0.006, *t* = 6.33, indicating the model with higher SR tends to produce more phonological SSEs.

**Table 2:** Linear mixed-effect model fitted to phonological SSE produced by the model.

	*Estimate β*	*t*	Confidence interval
WF	**−0.0102**	−19.07	(−0.0113, −0.0092)
ONS	**−0.0044**	−8.35	(−0.0054, −0.0034)
RC	**−0.0074**	−14.22	(−0.0084, −0.0064)
IMG	**−0.0035**	−6.7	(−0.0045, −0.0025)
SR	**0.0056**	6.33	(0.0039, 0.0074)
SR x RC	**−0.0023**	−13.08	(−0.0026, −0.0019)
SR x IMG	**−0.0015**	−8.44	(−0.0018, −0.0011)
RC x IMG	0.0001	0.22	(−0.0008, 0.001)
SR x RC x IMG	**−0.0007**	−4.15	(−0.001, −0.0003)

All predictors were scaled through *z*-score normalisation. Significant Estimate βs were marked in bold. WF: Word frequency; ONS: Orthographic neighbourhood size; RC: Rime consistency; SR: Semantic reliance; IMG: Imageability.

**Figure 4:**
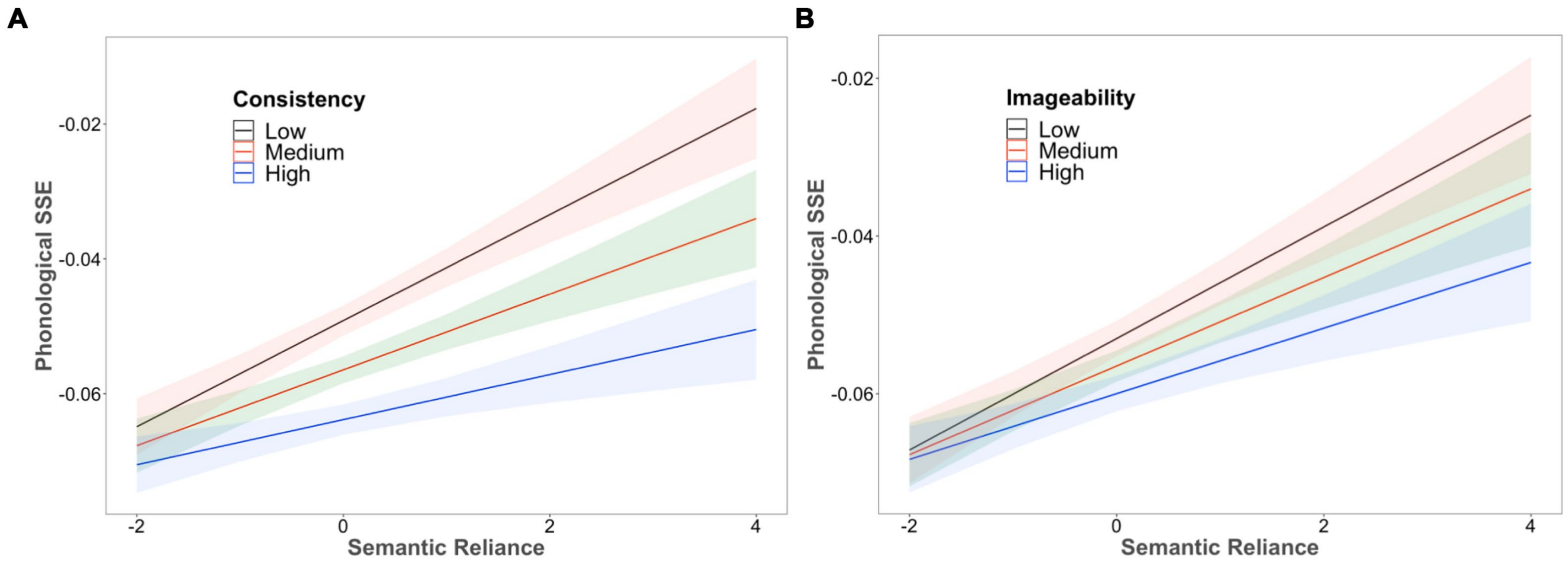
The interaction between **(A)** semantic reliance (SR) and rime consistency (RC) and **(B)** semantic reliance (SR) and imageability (IMG).

Regarding the interaction results, there were significant two-way interactions between SR and RC, *β* = −0.002*, t* = −13.08, and between SR and IMG, *β* = −0.002*, t* = −8.44, as shown in Figure 4. The model with a larger SR generated stronger consistency and imageability effects than that with a smaller SR. On the other hand, the interaction between RC and IMG was not significant. Critically, the three-way interaction between SR, RC and IMG also reached significance, *β* = −0.001*, t* = −4.15. Overall, these results are consistent with the findings of the factorial test of the SR effect reported in the previous section and the behavioral data (Woollams et al., 2016).

## 4 Discussion

The issue of effective early reading instruction has been a widely discussed topic in reading research (Rayner et al., 2001; Nation, 2009; Suggate, 2016; Taylor et al., 2017; Castles et al., 2018; Torgerson et al., 2019). Most studies have focused on investigating the benefits of using phonics-style or whole-word style when teaching children to read (e.g., Rayner et al., 2001; Nation, 2009; Davis, 2013; Taylor et al., 2017). However, little is known about the influence of early reading instructional methods on subsequent individual reading behaviors. Therefore, using a computational approach, we investigated whether early reading instruction could lead to variations among individuals’ sensitivity to OP and OS regularities, which in turn has an impact on reading behaviors. The simulation results demonstrated that the models receiving different training methods showed varying SR scores, displaying a spectrum of variations within each training condition (see Figure 2). On average, a model focused on print-to-meaning (the OS-focused training model) showed stronger SR for reading aloud than a model trained with a combination of OP and OS mappings (the OP-OS balanced training model), followed by a model focused on print-to-sound (the OP-focused training model).

Furthermore, by using both factorial and regression-based approaches, the LMM results indicated that the resulting SR scores were able to predict model performance on reading aloud. The models with higher SR scores tended to produce more phonological SSEs, compared to those with lower SR scores. The result was consistent with the findings of *slower* reading-aloud responses observed in behavioral studies (Siegelman et al., 2020, 2022) and *reduced* phonological activations observed in neuroimaging studies (Hoffman et al., 2015). Additionally, we also observed that the effect of SR interacted significantly with imageability and consistency as was reported in Woollams et al. (2016). Additionally, the LMM analyses revealed that several reading effects identified in behavioral studies (Balota et al., 2004; Cortese and Khanna, 2008), such as frequency, consistency, orthographic neighbourhood size, and imageability, could be accounted for by the model.

### 4.1 Reading instruction as a source of variations in individual differences

The simulation results, revealing diverse SR scores due to different reading instructional methods, align with our earlier modelling work (Chang et al., 2020), demonstrating the differential patterns of division of labour between OP and OS pathways as results of the models receiving different reading instructional methods. Our present study extends the finding by illustrating that the utilisation of the OP and OS pathways can be reframed as a measure of SR, thereby accounting for individual differences in reading.

The finding of varying degrees of utilisation of the OP and OS pathways relating to early reading instruction is potentially intriguing, particularly when considering the characteristics of English words. In early English word reading, approximately 80% of words are monomorphemic, based on token frequency (Rastle, 2019), and these words typically exhibit more systematic OP mappings compared to OS mappings (Plaut and Gonnerman, 2000). Thus, when the model learns the regularities of OP mappings of these words, the use of the direct OP pathway for a reading-aloud task should be mostly straightforward. Nevertheless, through intensive OS mapping training, there exists the potential for increased reliance on the OS pathway in the model. However, this enhancement may not be beneficial because the model may not be able to effectively exploit the systematic OP mappings, reflecting inferior performance in reading aloud for the OS-focused model compared to the OP-focused model. Considering rich evidence from behavioral and neuroimaging studies (Liberman et al., 1989; Simos et al., 2002, 2007; Castles et al., 2018; Rastle, 2019), a better practice may be to teach children OP mappings for developing phonological awareness in the early stages of reading instruction, followed by teaching OS mappings for integrating orthographic and morphological awareness.

Additionally, an important and relevant question is why the readers with high SR scores are slower in the reading-aloud task. Siegelman et al. (2022) suggest that an excessive reliance on the OS pathway might indicate an inadequate integrity of the phonological system, resulting in a suboptimal organisation of the reading system. According to contemporary neurocomputational models of language and reading processing (Hickok and Poeppel, 2004, 2007; Rauschecker and Scott, 2009; Ueno et al., 2011; Bornkessel-Schlesewsky and Schlesewsky, 2013), the dorsal and ventral pathways are involved with print-to-sound and print-to-meaning mappings, respectively. Thus, in the present context, one would anticipate observing distinctive modulations of neural activities in the dual pathways linked to the level of SR because of reading instruction. Indeed, neural activities in both pathways have been found to correlate with SR levels (Hoffman et al., 2015). Readers with higher SR scores display heightened activations in the left anterior temporal pole associated with semantic processing, and critically simultaneously exhibit reduced activations in the left precentral gyrus associated with phonological processing compared to those with lower SR scores. This neural evidence provides support for a potential explanation that high SR readers are slower in the phonologically-demanding task (Hoffman et al., 2015). This explanation aligns with Siegelman et al. (2022) account of a potential phonological deficit in the OP pathway in reading impaired children, reflected in increased SR. Our further investigations into the impact of reading instruction on learning in the model also provided computational evidence to support these accounts, demonstrating that the models with high SR scores produced lower phonological polarity scores and more phonological SSEs, especially for the OS-focused models.

Considering the broader implications for education, our simulation results suggest that some children entering intervention programmes already exhibit differences in their sensitivity to OP vs. OS regularities. The sensitivity differences contribute to divergent intervention outcomes, as demonstrated by Siegelman et al. (2022), wherein children relying more on OP regularities tend to show greater improvement in phonologically-weighted intervention programmes compared to those relying more on OS regularities. Interestingly, they also revealed that post-intervention, reading-impaired children who increased their reliance on OP regularities or decreased their reliance on OS regularities had better intervention gains. Their results highlight the importance of appropriate interventions to optimally shape OP and OS pathways in paving the way for reading success. Collectively, the results from computational modelling and behavioral investigations indicate that the type of training approaches used in early reading instruction and interventions can influence individuals’ reliance on the OP and OS pathways, contributing to individual differences in reading acquisition.

### 4.2 Limitations and future work

In this study, we have demonstrated a direct link between reading instruction and individual differences in reading by using a series of widely acknowledged triangle models of reading. Despite conducting a substantial number of simulations (i.e., 120 simulations) to capture variations in SR through an accelerated training process, this study could be enhanced by training additional simulation samples to achieve a scale similar to that of comprehensive behavioral studies (e.g., Siegelman et al., 2020, 2022) or to simulate dyslexic readers’ reading profiles (e.g., Perry et al., 2019).

Another notable aspect concerns the impact of reading instruction on language systems characterised by different forms of systematicity. The present study has focused on early English word reading, in which words generally exhibit more systematic OP mappings compared to OS mappings. Future investigations could explore language systems with a more balanced systematicity in the mappings between orthography to phonology and orthography to semantics, such as Chinese.

Lastly, the present study has focused on investigating the unique impact of early reading instruction on individual differences in reading, particularly regarding the utilisation of OP and OS pathways. However, literacy development is complex; various elements such as oral language knowledge, computational compacity, and reading experience may collectively contribute to different aspects of individual differences in reading (Plaut, 1997; Dilkina et al., 2008; Yap et al., 2012; Andrews, 2015; Siegelman et al., 2020; Chang, 2023). Therefore, in connecting to real-world applications, it is possible to extend the present simulation framework to incorporate and examine various potential factors comprehensively in future research. That could enable a better understanding of how to best tailor individual interventions and pave the way for the development of an intervention program suited to the diverse needs of beginning readers.

## 5 Conclusion

To conclude, this study utilised a large number of computational models of reading, developed using the optimised MikeNet simulator (see Supplementary material), to investigate the potential sources of individual differences in reading. The simulation results demonstrated a direct link between reading instruction and the variations in reliance on the OP and OS pathways within the model. This differential reliance significantly moderated model performance during reading.

## Data availability statement

The datasets presented in this study can be found in online repositories. The names of the repository/repositories and accession number(s) can be found below: https://github.com/yaningchang/Chang_et_al_RI_ID_Paper_for_FHN.git.

## Author contributions

Y-NC: Conceptualization, Data curation, Formal analysis, Funding acquisition, Investigation, Methodology, Project administration, Resources, Software, Supervision, Validation, Visualization, Writing – original draft, Writing – review & editing. T-JC: Methodology, Software, Supervision, Writing – original draft, Writing – review & editing. W-FL: Methodology, Software, Supervision, Writing – original draft. C-EK: Methodology, Software, Writing – original draft. Y-TS: Methodology, Software, Writing – review & editing. H-WL: Methodology, Software, Writing – review & editing.

## Footnotes

^1^We are grateful to reviewer 3 for expanding on this perspective.
