# Supplementary material for: Modelling individual differences in reading using an optimised MikeNet simulator: the impact of reading instruction
Source: Front Hum Neurosci. 2024 Jun 21;18:1356483. doi: 10.3389/fnhum.2024.1356483 (PMC11224532; doi:10.3389/fnhum.2024.1356483)
Supplement: Supplementary file 1 [file Table_1.DOCX]

### **Optimised MikeNet simulator**

### Considering computational modelling has proven to be a valuable tool for probing the mechanisms underlying language processes, training a large-scale deep neural network model, such as the fully implemented triangle model of reading employed in this study, is often associated with high computational costs. Consequently, the time-consuming training process fundamentally limits the model's capacity to simulate a substantial cohort of individuals. To mitigate the training burden and facilitate computational megastudies, optimising and parallelising the computationally demanding algorithms is imperative by leveraging available computing hardware resources. In this study, we integrated Single Instruction Multiple Data (SIMD) and threading optimisation techniques to enhance the computational performance and efficiency.

### **S1. Profiling and optimisation of simulation performance**

The present model was constructed using a MikeNet simulator developed by Harm and Seidenberg (2004). Although MikeNet is commonly used for building large-scale computational models of reading (Harm & Seidenberg, 2004; Monaghan et al., 2017), training such a model is generally computationally expensive and time-consuming. Extended training durations may stem from bottlenecks in the calculation of backpropagation errors and connections during weight updates. Therefore, to identify performance bottlenecks in the model constructed using the MikeNet simulator, we profiled the model's training process. Specifically, we used Valgrind (Weidendorfer et al., 2004), a widely-used open-source dynamic instrumentation framework, to conduct performance analysis of the present model and obtain the breakdown of time taken by each function throughout the entire computation process. Subsequently, to accelerate the training process, we then leveraged two high-performance C libraries, oneAPI Math Kernel Library (oneMKL) and Basic Linear Algebra Subprograms (BLIS), targeting the Intel and AMD processors, respectively. Both libraries contained optimised math routines commonly used for software performance optimisations in the science, engineering, and financial applications on x86 microprocessors. The libraries offer various subroutines, including basic linear algebra (BLAS), fast Fourier transforms (FFT), statistics, random number generation, and optimisation solvers.

### **S2. Simulator Optimisation**

As a test case, our initial simulator optimisation effort focused on profiling the oral language training phase during model training. Figure S1A delineates the functions consuming the majority of time during the training process of the model on the MikeNet simulator, along with their corresponding proportions of the overall training time. The analysis revealed that matrix-vector multiplication (matrix_vec_mult, matrix_vec_mult_t) and outer product (matrix_outer_product) constituted the major time-consuming operations, accounting for 38.7%, 28.4%, and 10.0% of the training time, respectively.

Figure S1. (A) The percentages of the execution time of different function calls in MikeNet in the training procedure. (B) The relative speedup of MikeNet with the different optimisation approaches.

As is evident from the profiling results presented in Figure S1A, matrix-vector multiplication significantly influenced the overall training time. Fortunately, this operation can be efficiently parallelised. The CPU employed in the simulation supports x86-64's single instruction multiple data (SIMD) extensions, enabling data-level parallelism (DLP) exploitation. We utilised the Intel oneMKL and AMD BLIS library to leverage the hardware's capabilities fully, incorporating supported SIMD intrinsics to enhance simulation performance. Importantly, these libraries offered BLAS APIs, providing a convenient solution for our requirements. Such that we could improve simulator performance by replacing the original matrix-vector multiplication implementation with cblas_sgemv() of oneMKL or bli_sgemv() of BLIS.

Table S1 details the elapsed training time of the oral language phase of the model, comparing instances with and without software optimisation using the Intel oneMKL library. In Figure S1B, the grey bar represents the training time of the unoptimised original MikeNet, while the middle bar illustrates the training time with the sequential version of Intel oneMKL. Exploiting the SIMD extension resulted in a significant reduction of more than 70% in the training time of MikeNet with optimisation, rendering a performance 3.7 times faster than the original.

Table S1. Training time and speedup ratio with/without SIMD optimisation

| Iterations | Training time without optimisation (s) | Training time with SIMD optimisation (s) | Speedup ratio |
| --- | --- | --- | --- |
| 200 | 2.35 | 0.64 | 3.67 |
| 500 | 5.77 | 1.65 | 3.49 |
| 1000 | 11.70 | 3.27 | 3.58 |
| 2000 | 23.76 | 6.43 | 3.69 |

Note: SIMD: Single instruction multiple data

To further enhance performance, we also investigated the impact of applying multithreading to the matrix-vector multiplication function in the MikeNet simulator. Specifically, the performance trend with varying numbers of instantiated threads was investigated and the experimental setup employed an Intel® Core™ i7-10700 CPU @ 2.90GHz processor with 8 cores and 2 threads per core. As in Figure S2, ‘baseline’ represents the unoptimised simulator, while 'sequential' corresponds to the SIMD-optimised version utilising a single thread for MKL functions internally. The result revealed that the best number of threads was 6, rendering a performance 4.8 times faster than the original in Figure S1B, and beyond that, an increase in training time became evident. Following the verification of the optimisation advantage in oral language training, we proceeded to train the complete reading model utilising the optimised MikeNet simulator.

Figure S2. The relative speedup of MikeNet with the different optimisation approaches.

### **S3. The use of an accelerated simulator**

The optimisation result demonstrated a notable speedup, achieving performance 4.8 times faster through both SIMD and threading optimisations compared to the original implementation. However, beyond 6 threads, an increase in training time becomes evident. Several potential reasons may account for this phenomenon. Firstly, performance degradation often occurs when the number of threads exceeds physical cores. Switching between threads introduces overhead due to state saving or lock holding before thread changes. Performance is hindered if a thread holds a lock, and other threads need to wait for it to release the lock upon its time slice expiration. Ideally, if thread switching can hide I/O wait delays, performance gains might be possible. However, excessive threading can hurt performance if this advantage fails to offset context switch overhead. Furthermore, a clear correlation exists between efficiency, thread count, and matrix sizes. Increasing thread count necessitates larger problem sizes to maintain comparable efficiency to smaller thread counts. For excessively small problem sizes, overhead may outweigh performance improvements. Additionally, if a function's computational intensity is low, and performance primarily hinges on memory access timings, invoking more threads may not enhance performance as memory bandwidth becomes saturated.

Moreover, the results of acceleration processes highlight the fact that multithreading does not always guarantee improved performance. Addressing issues such as synchronisation, data sharing, data alignment, and context switch overheads is crucial for future work aiming to achieve further performance enhancements.

### **Reference**

Harm, M. W., & Seidenberg, M. S. (2004). Computing the meanings of words in reading:

Cooperative division of labor between visual and phonological processes [Review].

*Psychological Review*, *111*(3), 662-720. <https://doi.org/10.1037/0033-295x.111.3.662>

Monaghan, P., Chang, Y.-N., Welbourne, S., & Brysbaert, M. (2017). Exploring the relations

between word frequency, language exposure, and bilingualism in a computational

model of reading. *Journal of Memory and Language*, *93*, 1-21.

<https://doi.org/https://doi.org/10.1016/j.jml.2016.08.003>

Weidendorfer, J., Kowarschik, M., & Trinitis, C. (2004, 2004//). A Tool Suite for Simulation

Based Analysis of Memory Access Behavior. Computational Science - ICCS 2004,

Berlin, Heidelberg.
